# Supplementary material for: Disruption of a DUF247 Containing Protein Alters Cell Wall Polysaccharides and Reduces Growth in Arabidopsis
Source: Plants (Basel). 2023 May 15;12(10):1977. doi: 10.3390/plants12101977 (PMC10221614; doi:10.3390/plants12101977)
Supplement: Supplementary file 1 [file plants-12-01977-s001.zip › Figure S1.pdf]

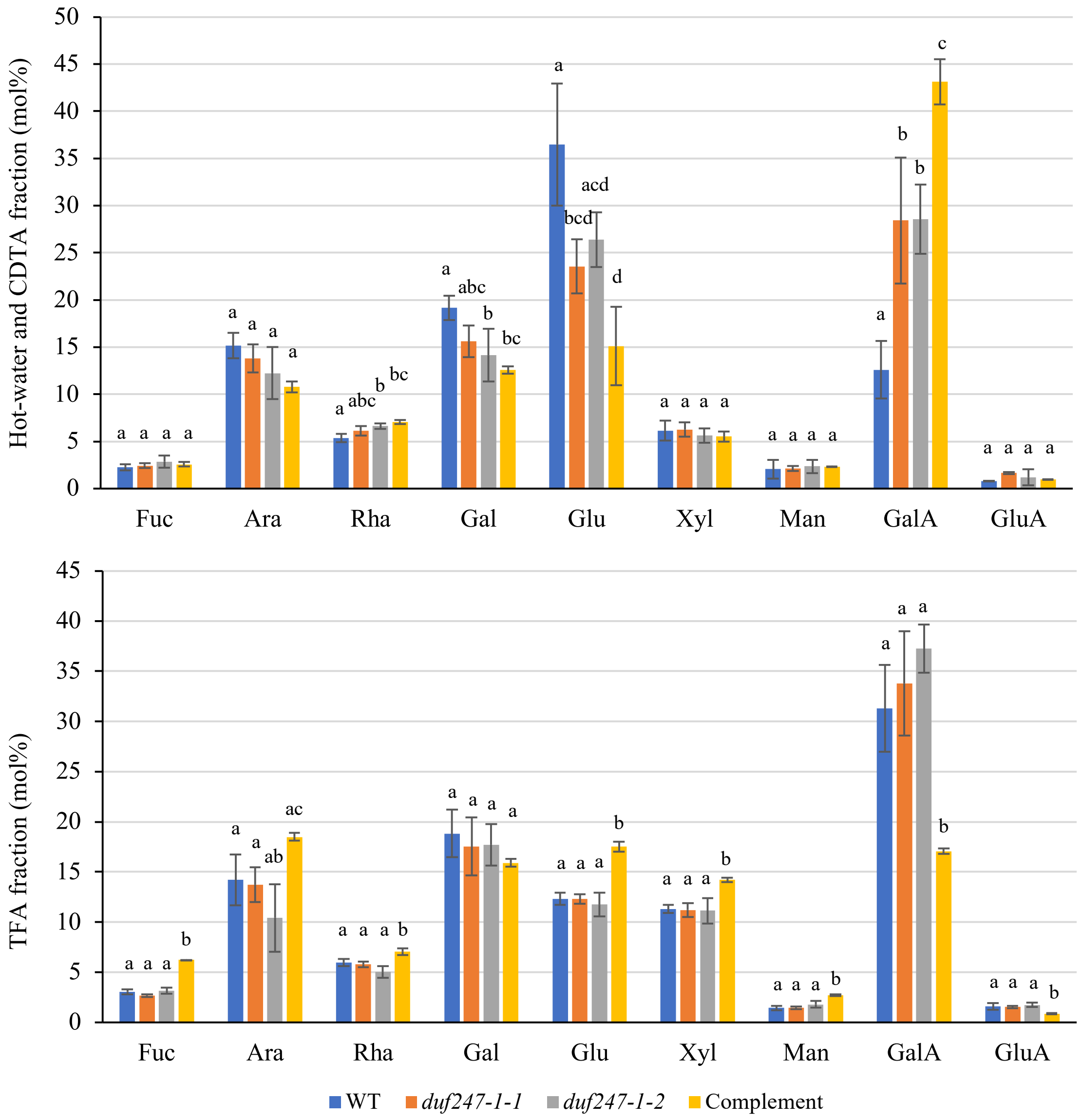

Figure S1. Monosaccharide compositions of hot water-CDTA and TFA fractions from seven day-old seedlings expressed in mol%. Data were obtained from three biological replicates presented with SE (n=3). Means with the same letter are not significantly different based on the Tukey's HSD test ($P < 0.05$).
