# Supplementary material for: Disruption of a DUF247 Containing Protein Alters Cell Wall Polysaccharides and Reduces Growth in Arabidopsis
Source: Plants (Basel). 2023 May 15;12(10):1977. doi: 10.3390/plants12101977 (PMC10221614; doi:10.3390/plants12101977)
Supplement: Supplementary file 1 [file plants-12-01977-s001.zip › Figure S2.pdf]

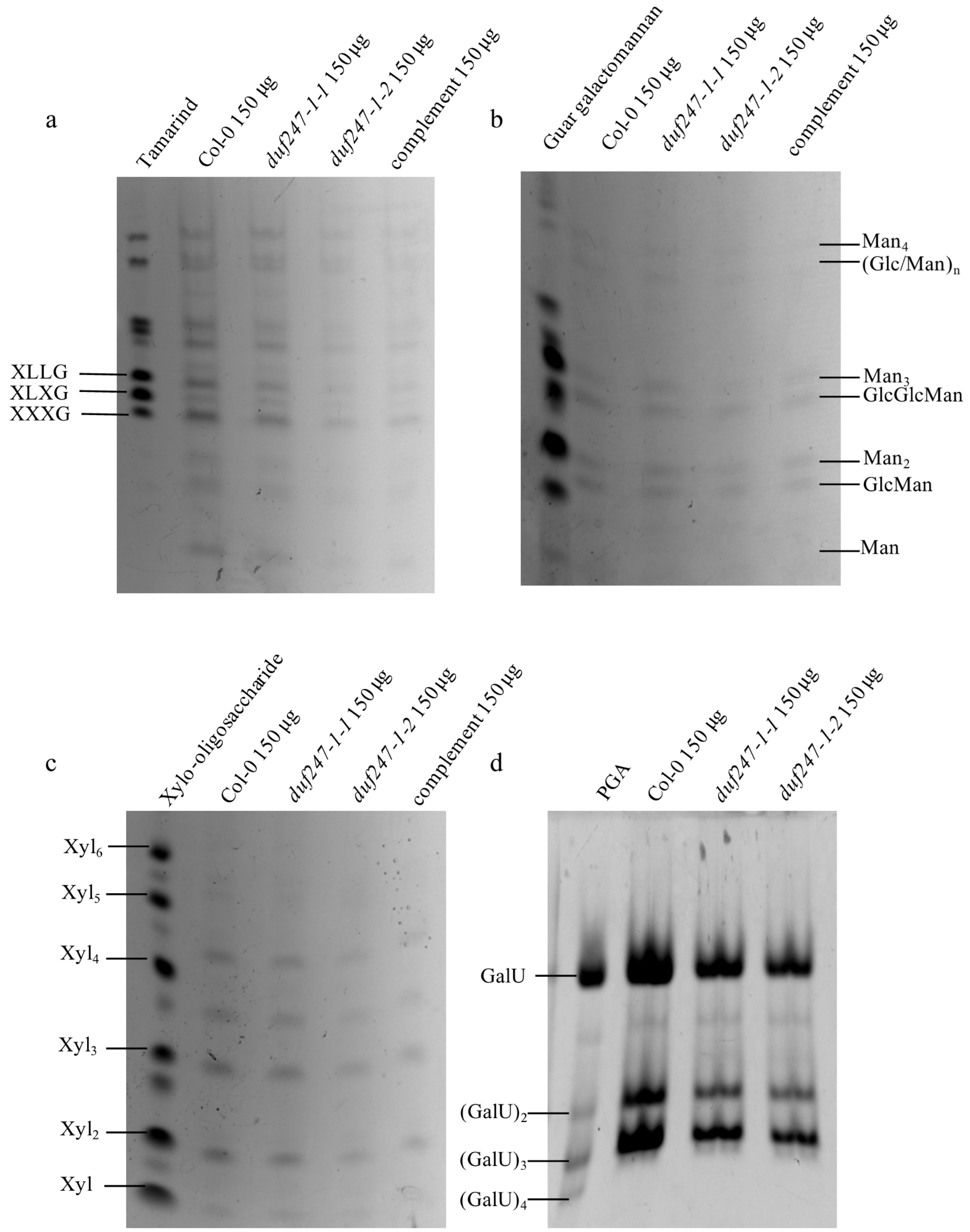

Figure S2. PACE analysis of NaOH extracts digested with endoglucanase II (a), mannanase (b) and xylanase (c) and CDTA extracts digested with polygalacturonase (d). Tamarind XyGs, xylooligosaccharides (DP1-6), 1,4-betaD-mannan, guar galactomannan and PGA were used as standard oligosaccharide markers. The identity of bands was based on co-migration patterns.
