# Supplementary material for: Disruption of a DUF247 Containing Protein Alters Cell Wall Polysaccharides and Reduces Growth in Arabidopsis
Source: Plants (Basel). 2023 May 15;12(10):1977. doi: 10.3390/plants12101977 (PMC10221614; doi:10.3390/plants12101977)
Supplement: Supplementary file 1 [file plants-12-01977-s001.zip › Tables S1-S2.pdf]

Table S1. Primers for PCR genotyping and vector constructions.

Primer	Sequence (5'-3' direction)
SAIL-LB	TTCATAACCAATCTCGATACAC
1252-F	TCACCAGATCCGATCAAGATC
1252-R	CCAGCCTCTTTTAGTTCCGTC
382-F	ATTGGGTGATCTCGATCACAG
382-R	CCACATCAATACATCATGATTCC
D247_2P-F	GTCAAGAAGTGGCTTTCGAC
D247_2P-R2	GATTTGAAGATATAATTGG
CDS-F	ATGGTGGCTGTCTTCTACAAAGAC
CDS-R	GTCCCGCGGTATACCACAAG
licCDS-F	ACGATACTCGAGGGGGATCCATGGTGGCTGTCTTCTAC
licCDS-FLAG-R	ACGATCGGGGAAATTCGCTAGTGGATCTTCACTTGTCGTCATCGTCT TTGTAGTCGGATCCAGAAGGTGGCTTATAATA
35S promoter-F	CGAATCTCAAGCAATCAAGC
NOS termi-R	AATCATCGCAAGACCGGCAAC

Table S2. Primers for qRT-PCR of the DUF247 family clade II.

Primer	Sequence (5’-3’ direction)
247-1-F	TGTGACGGAACTAAAAGAGGCTGGT
247-1-R	GAATCGCTCCCTAGCCAATGTTCG
247-2-F	CGCCATGAAAGAACTCGAAGACAGG
247-2-R	GGTGCCGTTTACCAAGCTGTATCTC
247-3-F	CGAGGTTATCGCGGAGTAGATGG
247-3-R	CGTTCGTTGAATCGATATGGCACT
247-4-F	CAGACGGTTGTCATGGTTCG
247-4-R	CCAGCTATTTGTTGCGTCG
247-5-F	CATGGAGCGTCACAAGTGG
247-5-R	GAATCCTTCACTTGTTCCCTTG
247-6-F	CTTGCTCAGCCTCAAGCTC
247-6-R	GAGATCACCCAACTGTCTCC
247-7-F	TCGAGCTCCTCCAAGGAGTCAATG
247-7-R	AGGCGAGTTCTCGGTCAACGTC
247-8-F	CAGCTACCATTTGGACCGAAG
247-8-R	GCGTCTCTCATTACTTGCTCC
247-9-F	CGATCAAGCACATAGAGACGAC
247-9-R	GTAGGATTAGACTGGAGCAGAC
ACTIN-F	ACTCTCCCGCTATGTATGTC
ACTIN-R	GATGGAAGAGCTGGTCTTTG
UBQ10-F	TTTGTTAAGACTCTCACCGGAAAGACA
UBQ10-R	GAGGGTGGATTCCTTCTGGATATTGTA
