# Supplementary figures and images for: Disruption of a DUF247 Containing Protein Alters Cell Wall Polysaccharides and Reduces Growth in Arabidopsis
Source: Plants (Basel). 2023 May 15;12(10):1977. doi: 10.3390/plants12101977 (PMC10221614; doi:10.3390/plants12101977)

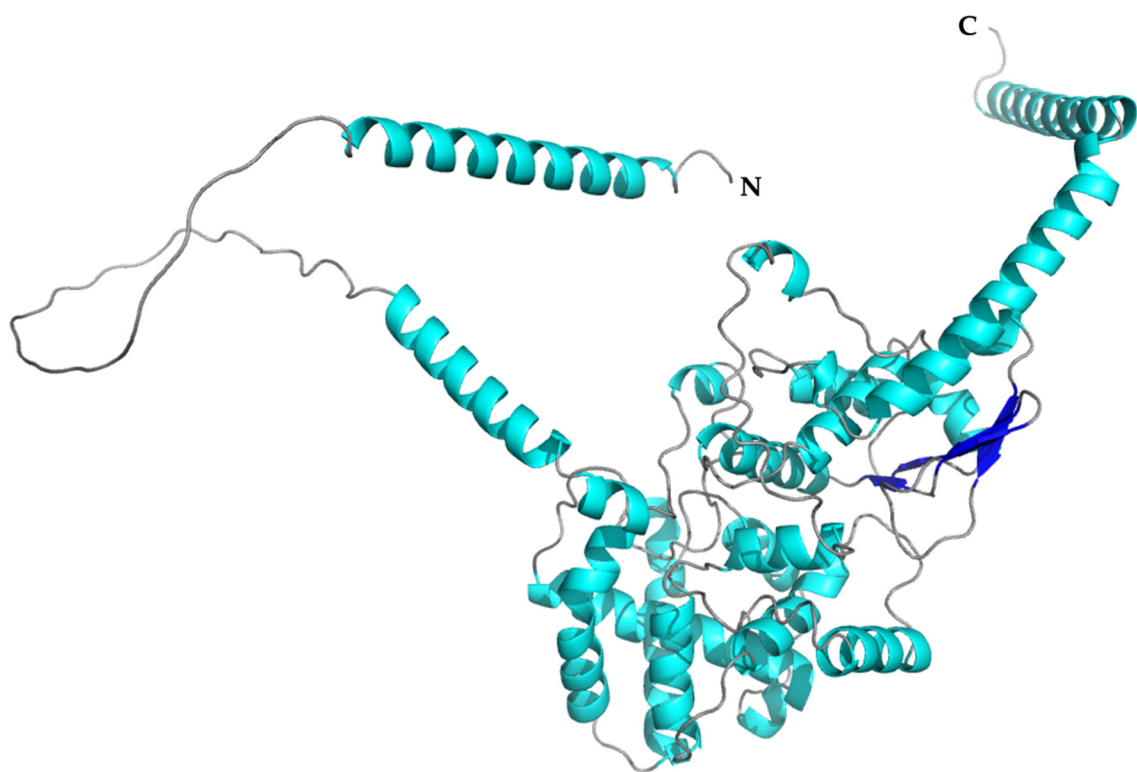

1

2

3 Figure S3. Predicted structure of DUF247-1 from AlphaFold Protein Structure Database.

4

Supplement: Supplementary file 1 [file plants-12-01977-s001.zip › Figure S3.pdf]
